# Oleate of Mercury

**Published:** 1873-11

**Authors:** 


*THE LONDON LANCET:*

Oleate of Mercury.—The advantages which oleate of mercury seem to possess over other remedies are:

1. It is a certain remedy if carefully applied.

2. It produces no staining or injury of the skin. In cases where the disease appears on the face, it is of great importance to avoid any disfigurement or staining.

3. It is painless in its application. This is not the case with the ordinary strong parasiticides, most of which produce vesication, etc.

4. It readily penetrates into the sebaceous glands, hair-follicles, and even into the hairs themselves, the mercury being in a state of solution in an oily medium, and it is therefore much more likely to destroy the fungus than the spirituous or acqueous solutions of mercury, etc. This penetrating power of the oleate may be increased by adding a small quantity of ether (one part to eight) to it.

In very sensitive skins the irritation sometimes produced by it may be avoided by using a weaker solution, (five per cent.), and by applying it with a camel's-hair brush. In slight cases this method is all that is necessary, but where the fungus has invaded the hair it is advisable to rub in the oleate gently.

